# Supplementary material for: The Antibiotic Fosfomycin Mimics the Effects of the Intermediate Metabolites Phosphoenolpyruvate and Glyceraldehyde-3-Phosphate on the Stenotrophomonas maltophilia Transcriptome
Source: Int J Mol Sci. 2021 Dec 23;23(1):159. doi: 10.3390/ijms23010159 (PMC8745565; doi:10.3390/ijms23010159)
Supplement: Supplementary file 1 [file ijms-23-00159-s001.zip › ijms-1487426-supplementary.pdf]

A

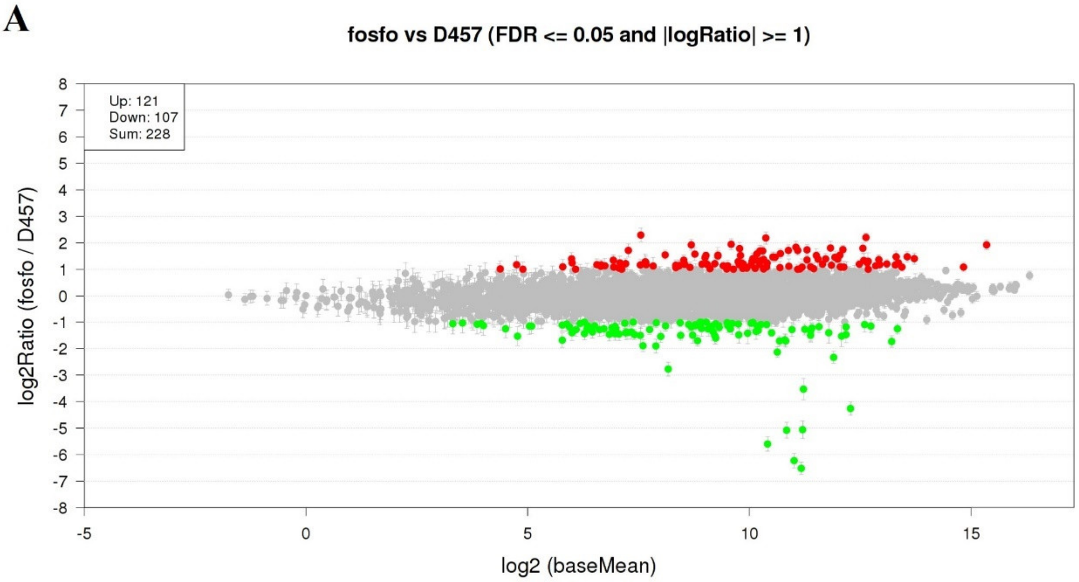
fosfo vs D457 (FDR <= 0.05 and |logRatio| >= 1)
Up: 121
Down: 107
Sum: 228
log2Ratio (fosfo / D457)
log2 (baseMean)

B

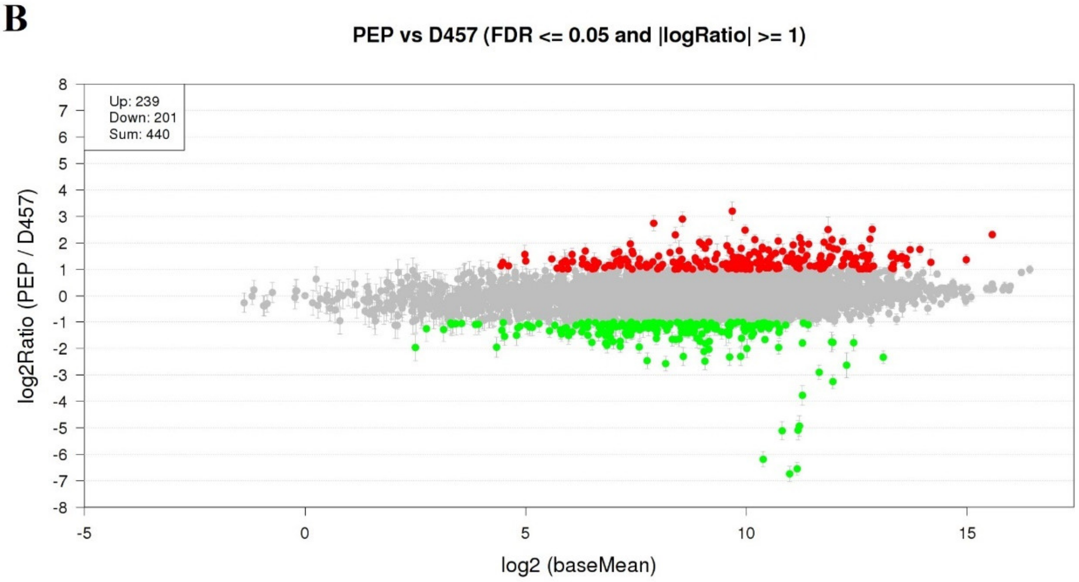
PEP vs D457 (FDR <= 0.05 and |logRatio| >= 1)
Up: 239
Down: 201
Sum: 440
log2Ratio (PEP / D457)
log2 (baseMean)

C

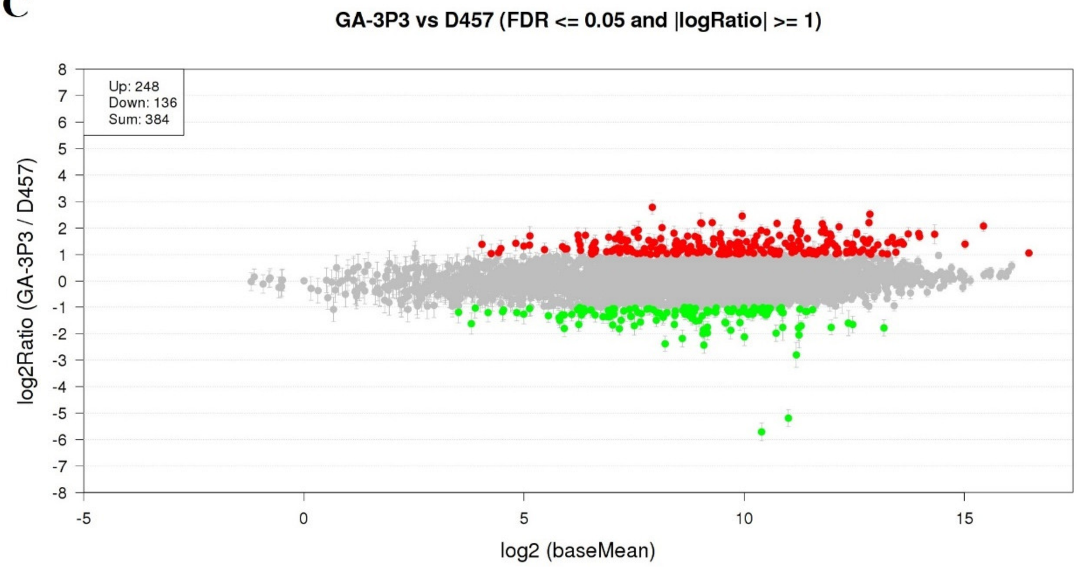
GA-3P3 vs D457 (FDR <= 0.05 and |logRatio| >= 1)
Up: 248
Down: 136
Sum: 384
log2Ratio (GA-3P3 / D457)
log2 (baseMean)

Figure S1. Differential expression between sample groups and their statistical significance. A) Fosfomycin; B) PEP and C) GA-3P. Fold changes were calculated as log_2 of RPKM after the treatment in comparison with the RPKM of the wild-type D457 strain without treatment. Fold changes <-1 or > 1 were considered significant.

Table S1. List of putative *S. maltophilia* D457 T4SS effectors* identified by BLAST search using XVIPCD domains from X. citri effectors.

X. citri XVIPCD	*S. maltophilia* D457											
Entry	Entry/E-value											
XAC4264 (140-279)	SMD_RS10655<br>6,0E-13	SMD_RS13930<br>2,0E-09	SMD_RS13920<br>5,0E-08									
XAC3634 (189-306)	SMD_RS01005<br>3,0E-16	SMD_RS13735<br>5,0E-16	SMD_RS01500<br>2,0E-15	SMD_RS03560<br>3,0E-14	SMD_RS13725<br>9,0E-11	SMD_RS10655<br>5,0E-10	SMD_RS02370<br>4,0E-08	SMD_RS13930<br>1,0E-04	SMD_RS00480<br>2,0E-04	SMD_RS02350<br>4,0E-04	SMD_RS13920<br>2,0E-03	SMD_RS12385<br>5,6E+00
XAC3266 (735-861)	SMD_RS14045<br>5,7E+00											
XAC2885 (271-395)	SMD_RS13735<br>2,0E-12	SMD_RS01005<br>5,0E-08	SMD_RS13920<br>1,0E-06	SMD_RS13930<br>3,0E-06	SMD_RS01500<br>3,0E-05							
XAC2609 (315-431)	SMD_RS01500<br>7,0E-27	SMD_RS03560<br>2,0E-21	SMD_RS13735<br>1,0E-20	SMD_RS01005<br>2,0E-16	SMD_RS13725<br>2,0E-11	SMD_RS00480<br>2,0E-06	SMD_RS02350<br>3,4E-02					
XAC1918 (477-606)	SMD_RS13920<br>5,0E-25	SMD_RS13930<br>5,0E-25	SMD_RS10655<br>3,0E-19	SMD_RS02370<br>2,0E-18	SMD_RS02340<br>4,0E-16	SMD_RS13735<br>3,0E-07	SMD_RS00480<br>7,0E-07	SMD_RS02350<br>1,0E-06	SMD_RS01500<br>8,0E-06	SMD_RS03560<br>9,0E-03		
XAC1165 (1-112)	SMD_RS02370<br>5,0E-28	SMD_RS13930<br>8,0E-26	SMD_RS10655<br>1,0E-24	SMD_RS13920<br>5,0E-24	SMD_RS02340<br>4,0E-18	SMD_RS02350<br>3,0E-13	SMD_RS13725<br>3,0E-11	SMD_RS00480<br>1,0E-10				

XAC0574<br>(317-440)	SMD_RS13<br>725<br>3,0E-44	SMD_RS10<br>655<br>5,0E-15	SMD_RS13<br>930<br>1,0E-14	SMD_RS13<br>920<br>1,0E-12	SMD_RS13<br>735<br>1,0E-12	SMD_RS01<br>005<br>5,0E-12	SMD_RS02<br>370<br>1,0E-11			
XAC0466<br>(488-584)	SMD_RS14<br>175<br>2,0E-18	SMD_RS14<br>175<br>9,0E-14								
XAC0323<br>(16-136)	SMD_RS02<br>370<br>1,0E-25	SMD_RS10<br>655<br>1,0E-22	SMD_RS02<br>340<br>2,0E-22	SMD_RS13<br>930<br>1,0E-21	SMD_RS13<br>920<br>1,0E-20	SMD_RS00<br>480<br>1,0E-11				
XAC0151<br>(120-254)	SMD_RS02<br>370<br>3,0E-36	SMD_RS10<br>655<br>8,0E-32	SMD_RS13<br>930<br>1,0E-29	SMD_RS13<br>920<br>2,0E-29	SMD_RS02<br>340<br>9,0E-29	SMD_RS02<br>350<br>4,0E-18	SMD_RS00<br>480<br>2,0E-15	SMD_RS13<br>735<br>3,0E-11	SMD_RS13<br>725<br>2,0E-06	SMD_RS03<br>560<br>2,0E-05
XAC0096<br>(506-646)	SMD_RS10<br>655<br>4,0E-21	SMD_RS13<br>930<br>5,0E-20	SMD_RS13<br>920<br>1,0E-16	SMD_RS02<br>370<br>2,0E-16	SMD_RS02<br>340<br>6,0E-15	SMD_RS13<br>725<br>1,0E-09	SMD_RS13<br>735<br>5,0E-09	SMD_RS02<br>350<br>1,0E-06	SMD_RS01<br>500<br>6,0E-05	SMD_RS03<br>560<br>7,0E-03

*Effectors are color coded according to their accession number and organized according to the e-value.

Table S2 Primers used in this study

Primer	Sequence (5´- 3´)	Description
27	TGCCAGCGACAGTGCAAAGGGTC	Amplification of *smeT* to test DNA contamination
48	CCGTGTTCATGGAAGCAGGC	
gyrA_F	CGCAAGAGXTACCTCGATTA	To amplify the housekeeping gene *gyrA* by real-time PCR
gyrA_R	GGTGGTACTTACCGATGACG	
virB2_F	AATCTGGTAGCCAGCAAAGA	To amplify *virB2* by real-time PCR
virB2_R	ACGCTGAAGACTCTGCTGAT	
virB8_F	CCAGCTGGTTGTCAAGGTAG	To amplify *virB8* by real-time PCR
VirB8_R	GTAACCCTCAGAACCCGTTT	
virB9_F	CAGTACAAGATCGCCTTCGT	To amplify *virB9* by real-time PCR
virB9_R	GAAACGACCATCGTCATAGG	
virB10_F	GTCCAAAATGTCTCCAATGC	To amplify *virB10* by real-time PCR
virB10_R	ATAGATCTTGGAGCCCTTCG	
virD4_F	TCAAGCAGGAGAACTTCGAC	To amplify *virD4* by real-time PCR
virD4_R	TACAGGGTAAAGGCCATGAA	
fruK_F	AACATCAAGCTGGTGGAAGA	To amplify *fruK* by real-time PCR
fruK_R	GCCACTGGTATCCAGCAGTA	
fruA_F	TCGGCCTGTTGATGATGTAT	To amplify *fruA* by real-time PCR
fruA_R	GGAAATCAATGCGGTAGAG	
rpfN_F	TGATCGAGGATCATTTCCTG	To amplify *rpfN* by real-time PCR
rpfN_R	CCATAGCTCAGCTCCATCAT	
dld_F	GTTCCACCAGGACTACATCG	To amplify *dld* by real-time PCR
dld_R	CCTTTTTCCTTCGAGGTCTG	

Primer	Sequence (5´- 3´)	Description
lctD_F	GACCTGAGCCTGGAAACC	To amplify *lctD* by real-time PCR
lctD_R	ACGTACAGCTGGAACCACAT	
lldR_F	GAATACCGTTTTGACGTGCT	To amplify *lldR* by real-time PCR
lldR_R	CGTCAGCCTCTGCTTCAC	
lctP_F	GTTCGGATACCTCGTCCAAT	To amplify *lctP* by real-time PCR
lctP_R	GTGAAAATCAGGCTGTGCTT	
smeY_F	CATTGGTGACCGAAGGTG	To amplify *smeY* by real-time PCR
smeY_R	TTGATACCGGAGAACAGCAG	
smeZ_F	TGTACGAGAGCTGGTCGATT	To amplify *smeZ* by real-time PCR
smeZ_R	GAACTCGACAATGAGGATCG	
oprO_F	GAACAAGGATGACACCGAG	To amplify *oprO* by real-time PCR
oprO_R	TCCAGCGAGAAATACTGCTT	
